# The Post-Pandemic Return of *Mycoplasma pneumoniae*: Why Children Matter and What Clinicians Should Know

**DOI:** 10.3390/jcm15041644

**Published:** 2026-02-22

**Authors:** Takeshi Saraya

**Affiliations:** Department of Respiratory Medicine, Kyorin University School of Medicine, Tokyo 181-8611, Japan; sara@yd5.so-net.ne.jp; Tel./Fax: +81-(0)4-2244-0671

**Keywords:** *Mycoplasma pneumoniae*, post-COVID-19 resurgence, macrolide resistance, pediatric reservoir, respiratory viral co-infection

## Abstract

*Mycoplasma pneumoniae* infections declined sharply during COVID-19 non-pharmaceutical interventions but have resurged in multiple regions after restrictions were lifted. This narrative Perspective synthesizes recent epidemiologic and molecular observations and emphasizes children as a key reservoir sustaining community transmission. We highlight how asymptomatic carriage and prolonged upper-airway detection complicate interpretation of PCR results, and we discuss the clinical implications of macrolide-resistant *M. pneumoniae* (MRMP), including pragmatic, age-stratified management considerations. Finally, we outline priorities for surveillance and for translating genomic signals into simplified clinical pathways, particularly in high-MRMP settings.

## 1. Introduction: Epidemiology—From Silence to Resurgence

*Mycoplasma pneumoniae* (Mp) is a common cause of respiratory tract infections in both children and adults. However, the non-pharmaceutical interventions (NPIs) implemented during the COVID-19 pandemic drastically reduced the transmission of *M. pneumoniae*. The global detection rate decreased from 8.61% in the pre-pandemic period (April 2017–March 2020) to 1.69% in the first year after NPI implementation (April 2020–March 2021) [1], 0.70% in the second year (April 2021–March 2022) [2], and 0.82% in the third year (April 2022–March 2023) [3].

Although levels remained low, an increase in case numbers was noted in several countries during the final months of the third year (January–March 2023) [3]. In the fourth year following the introduction of NPIs (1 April–30 September 2023), the ESGMAC MAPS study, performed at 45 sites across 24 countries in four UN regions (Europe, Asia, the Americas, and Oceania), demonstrated the re-emergence of *M. pneumoniae* in Europe and Asia [4]. Soon after this report, the WHO released a statement regarding clusters of *M. pneumoniae* infections among children in northern China [5].

Further global surveillance (65 sites in 29 countries) from 1 April 2017, to 31 March 2024, demonstrated that the PCR detection rate of *M. pneumoniae* rose to 11.47% (SD 15.82) during the re-emergence period (April 2023–March 2024) [6]. Macrolide-resistant *M. pneumoniae* (MRMP) rates in Europe and Asia were 2.02% and 71.22%, respectively, with no major differences between the re-emergence and pre-COVID-19 periods.

The time-series susceptible–infected–recovered (TSIR) model, assuming a 3-week generation time and a 90% reduction in transmission due to NPIs, accurately predicted the delayed re-emergence [6].

In the fifth year following the initial implementation of NPIs (1 April 2024–31 March 2025), continued surveillance across 68 sites in 32 countries showed mean PCR detection rates of 12.00% (SD 13.80) in Europe, 31.48% (SD 26.67) in Asia, 5.48% (SD 2.62) in the Americas, and 4.81% (SD 4.83) in Oceania. During the study period, MRMP rates reported by 17 sites averaged 3.02% (SD 2.97) in Europe (France, Belgium, England, Wales, and Italy), 60.90% (SD 21.12) in Asia (China, South Korea, Japan, Taiwan, and Afghanistan), and 36.46% (SD 36.82) in the Americas (the USA and Cuba) [7].

Thus, although NPIs substantially suppressed *M. pneumoniae* transmission, a widespread global resurgence became evident after pandemic restrictions were lifted. A recent global surveillance and transmission-modelling study has comprehensively described the spatiotemporal re-emergence of *M. pneumoniae* after COVID-19 NPIs across multiple regions, showing widespread increases in detections in late 2023 without an overall signal of markedly increased severity at the population level [6,7]. Several explanations have been proposed for the post-pandemic resurgence of *M. pneumoniae* (i) reduced population exposure during NPIs leading to a larger susceptible cohort (“immunity debt”) [8]; (ii) rapid restoration of close-contact mixing after reopening, particularly among school-aged children [9]; and (iii) re-emergence of cyclical epidemic behavior that may have been transiently suppressed during NPIs [10], potentially interacting with changes in predominant circulating strains/lineages.

Available data on macrolide-resistant *M. pneumoniae* (MRMP) are geographically uneven. In a global systematic review and meta-analysis, the pooled proportion of MRMP was highest in the WHO Western Pacific region (53.4%), whereas it remained lower in the Americas (8.4%) and Europe (5.1%), and was lowest in the Eastern Mediterranean region (1.4%); notably, no published data were identified from countries in the WHO African region [11].

## 2. Pathophysiology and Disease Mechanisms of *Mycoplasma pneumoniae*

**Figure 1:**
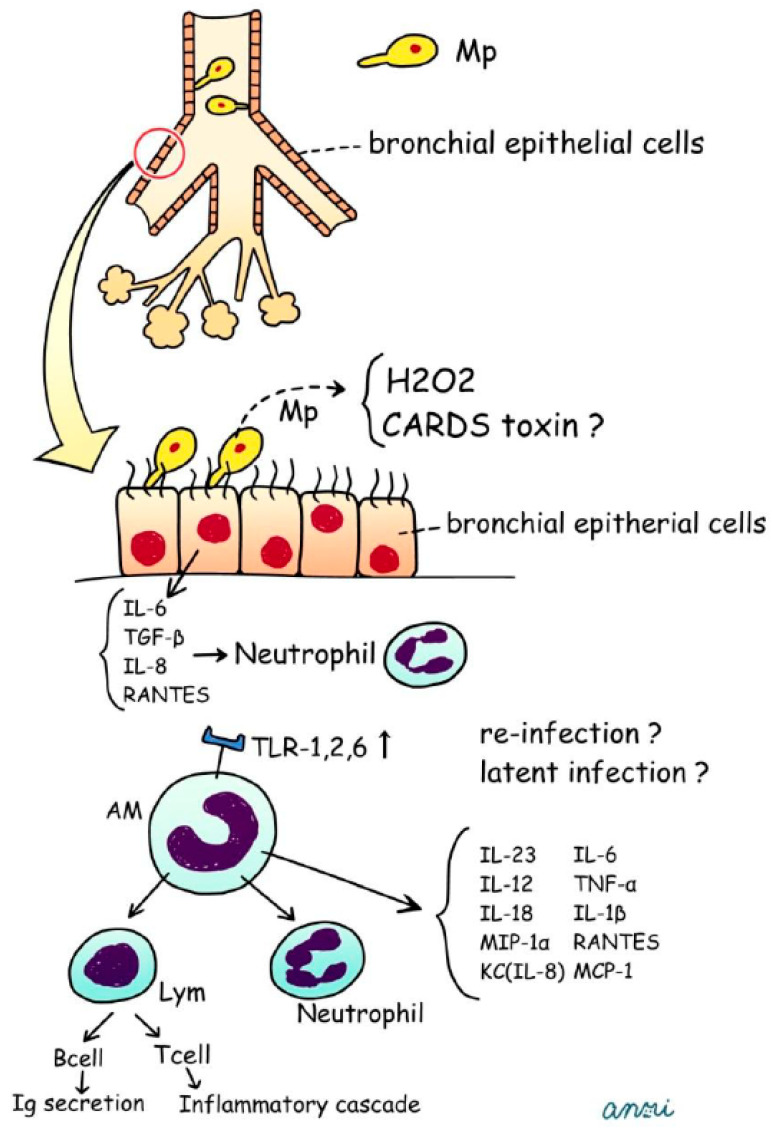
Postulated schema for pathogenesis of human Mp pneumonia. CARDS, Community Acquired Respiratory Distress Syndrome; TNF, tumor necrosis factor; RANTES, regulated on activation; normal T cell expressed and secreted; MCP-1, monocyte chemotactic protein-1. Reproduced with permission from Ref. [12].

First, *Mycoplasma pneumoniae* (Mp) attaches to bronchial epithelial cells and produces soluble hemolysin, hydrogen peroxide (H2O2), and superoxide radicals, all of which induce oxidative stress and lead to both structural and functional deterioration of cilia (Figure 1) [12].

The community-acquired respiratory distress syndrome (CARDS) toxin is present on the M. pneumoniae cell membrane, including at the tip organelle, in a distribution pattern similar to that of the P1 adhesin. CARDS toxin is thought to contribute to adherence in conjunction with the tip organelle, thereby facilitating intimate Mp–host interactions. In the lung, Mp-derived oxidants (H2O2) and CARDS toxin drive epithelial injury and excessive inflammatory responses. CARDS toxin is an established virulence factor with cytotoxic [13,14,15] and immunomodulatory effects [16,17]. In contrast, immune evasion through antigenic variation is primarily attributed to variability in surface-exposed adhesins/antigens; any synergistic interaction between CARDS toxin–driven inflammation and antigenic variation in persistence remains plausible but incompletely defined. Recently, Fang et al. described the establishment of a LAMP method for the rapid, straightforward, sensitive, and specific detection of CARDS toxin in Mp pneumonia [18]. Alveolar macrophages (AMs), which constitute approximately 93% of all pulmonary macrophages, serve as key innate immune effector cells during Mp infection [19]. Although partly derived from circulating monocytes, local proliferation contributes minimally to their overall numbers.

Mp-derived lipoproteins activate inflammatory signaling through TLR-2, and Mp is primarily recognized via TLR-1, TLR-2, and TLR-6 on AMs. Experimental models have shown that Mp infection increases TLR-2 expression and enhances recruitment of the adaptor protein MyD88, thereby amplifying downstream inflammatory responses.

Findings from germ-free, gnotobiotic, and conventional mouse models demonstrate that prior exposure—whether to live Mp or its antigens—primes the host for an augmented response upon re-exposure. Repeated antigenic stimulation upregulates TLR-2 expression on both alveolar macrophages (AMs) and bronchial epithelial cells, suggesting that even subclinical Mp exposure may “precondition” the lower airways. In this state, AMs become hypersensitive to subsequent encounters with Mp with innate immune responses being accelerated through TLR-2 upregulation induced by antecedent antigenic stimulation [19].

Activated AMs secrete a broad array of cytokines and chemokines, including IL-6, TNF-α, IL-1β, IL-18, MIP-1α, KC, RANTES, IL-12, IL-23, and MCP-1, which collectively drive the neutrophilic inflammation characteristic of Mp pneumonia.

## 3. Children as a Key Reservoir for *Mycoplasma pneumoniae* Transmission

Because children frequently carry *M. pneumoniae* in the upper airway—including in asymptomatic states—pediatric transmission networks likely seed and shape the outbreak trajectories that become visible later in broader community surveillance. In contrast, across adult cohorts, evidence supporting persistent or “latent” *M. pneumoniae* detection outside pneumonia has been limited [20]. Accordingly, in adults, *M. pneumoniae* is rarely identified in respiratory syndromes other than community-acquired pneumonia [21], supporting the hypothesis that children, rather than adults, constitute the principal reservoir sustaining transmission.

Spuesens et al. [22] reported that *M. pneumoniae* DNA in the upper respiratory tract could be detected by real-time PCR in both symptomatic (16.2%) and asymptomatic (21.2%) children with upper respiratory tract infection, suggesting that carriage of *M. pneumoniae* in young children can occur across a wide age range from 3 months to 16 years, which can persist for up to 4 months in a subset of children.

In a Dutch household contact study conducted in the pre–COVID-19 period, Dorigo-Zetsma et al. detected *M. pneumoniae* by PCR on nose–throat swabs in 15% of family contacts; three-quarters of these PCR-positive contacts were <16 years of age, and 44% did not develop acute respiratory infection, leading the authors to conclude that “apparently, children are a relevant reservoir for *M. pneumoniae*” [23]. During an outbreak of *M. pneumoniae* –associated Stevens–Johnson syndrome, Francois Watkins et al. investigated 15 household contacts, of whom 5 (33%) were *M. pneumoniae* positive; all but one (aged 51 years) were younger than 20 years, further underscoring the central role of children and adolescents in household transmission [24].

More recently, Koenen et al. reported that “while *M. pneumoniae* carriage can be asymptomatic, it forms a reservoir for family transmission and can precede *M. pneumoniae* infection” in children with recurrent respiratory tract infections [25]. Of note, the mean prevalence of upper respiratory tract carriage of *M. pneumoniae* was 53%, and the probability of being a carrier increased with the number of carrier household members: 26% when there were no positive household members, 36% when one member was positive, 41% when two members were positive, and 77% when three or more household members were carriers. Taken together, these data consistently indicate that school-aged children and younger children represent the main reservoir sustaining community and household circulation of *M. pneumoniae*.

This concept of children as the primary reservoir of *M. pneumoniae* has gained renewed prominence in the post-COVID era. Longitudinal sampling studies show that upper-airway detection of M. pneumoniae in children can persist for weeks and, in some cases, up to several months [22], complicating the distinction between carriage and symptomatic infection. Co-detection of respiratory viruses is common during pediatric respiratory illness, and it is plausible that viral co-infections may influence symptom expression and ongoing detection, although the causal relationship remains to be defined. From a public health perspective, these observations mean that school-aged children are not merely “victims” of the current resurgence but the engine that sustains transmission cycles. Consequently, interventions such as timely diagnosis in pediatric clinics, targeted education of families and schools, and judicious use of macrolides in this age group are likely to yield benefits that extend far beyond pediatrics and reduce the downstream burden of adult pneumonia.

## 4. Diagnostic Challenges and Opportunities

Culture remains the traditional reference (“gold standard”) method for diagnosing *Mycoplasma pneumoniae* (Mp) infection and is indispensable for downstream analyses such as antimicrobial susceptibility testing; however, it requires prolonged incubation in PPLO medium (often several weeks), limiting its utility for acute clinical decision-making. Serological assays are widely available, and Mp infection is often inferred from complement fixation (CF) titers of ≥1:64 or ≥1:128 or passive agglutination (PA) titers of ≥1:320 or ≥1:640 [26]. Nevertheless, because serologic responses are time dependent, confirmation of acute Mp pneumonia is generally strengthened by direct detection methods.

Accordingly, nucleic-acid amplification tests (NAATs) that detect mycoplasmal DNA are preferred for timely confirmation, including standard PCR-based assays as well as simplified formats such as loop-mediated isothermal amplification (LAMP) and quenching probe PCR (Q-probe PCR) [26]. Rapid antigen-based immunochromatographic assays have also been developed as point-of-care options, although confirmatory testing may be required when clinical suspicion remains high. More recently, multiplex PCR panels capable of simultaneously detecting Mp together with a broad range of respiratory viruses and bacteria have become increasingly adopted in clinical practice, particularly when co-infection is a concern or when an etiologic diagnosis is unclear. However, despite their advantages, molecular platforms have historically been concentrated in selected hospitals because of costs and the need for specialized equipment and workflows. Table 1 summarizes the typical turnaround time, key advantages and limitations, and recommended clinical scenarios for each diagnostic modality.

## 5. Major Regional Outbreaks of *Mycoplasma pneumoniae* (2023–2025)

In line with the above reservoir concept, age-stratified data from recent outbreaks [28,29,30,31,32,33,34] suggest that epidemics may be “child-led,” with a subsequent shift in disease burden toward adults (Table 2). Most recent reports from China and Denmark have described marked surges of *M. pneumoniae* infection predominantly among children [28,29,30,31]. Of note, in a large-scale outbreak in Marseille, Edouard et al. observed that children <15 years were more affected during the 2023–2024 epidemic than in previous seasons; however, they noted “a switch regarding the population affected by the epidemic,” as adults became more affected from January 2024 onward, “possibly because of a massive transmission of the bacterium from infected children” [32]. These studies also demonstrated marked regional differences in the prevalence of macrolide-resistant *Mycoplasma pneumoniae* (MRMP) (Table 2).

The 2023 resurgence of *Mycoplasma pneumoniae* in China was driven by two macrolide-resistant epidemic clusters, T1-2-EC1 and T2-2-EC2 [35]. All isolates within these clusters carried the 23S rRNA A2063G mutation, conferring high-level macrolide resistance. Importantly, these clusters did not represent newly emerging variants; rather, T1-2-EC1 can be traced back to approximately 1997, and T2-2-EC2 appears to have arisen around 2014, with both gradually expanding and eventually predominating during the 2023 epidemic. Macrolide prescribing patterns may also partly explain the high MRMP prevalence in mainland China. Following the 2007 pediatric guideline recommending azithromycin as the preferred first-line macrolide, ecological and temporal associations were reported between increased azithromycin use and rising MRMP rates in some regions [36]. By contrast, Outside Asia, in Ohio, MRMP prevalence was reported to decline toward baseline as azithromycin prescribing decreased after epidemic periods [37], consistent with strong population-level selection pressure.

Strikingly, the genotype dynamics observed in Japan and China during the post-pandemic resurgence suggest a shared regional evolutionary trajectory, rather than independent or contradictory national trends. In China, the 2023 epidemic reflected the nationwide re-expansion of the macrolide-resistant T1-2-EC1 lineage, which has increasingly dominated the circulating *M. pneumoniae* population. One year later, Japan exhibited a similarly notable pattern: an abrupt shift from P1 type 2—the predominant genotype during the previous epidemic—to an overwhelming predominance of P1 type 1 in 2024 [34].

Rather than indicating divergent selective pressures, these parallel transitions are consistent with a broader “type 1 re-emergence” across East Asia, likely shaped by a combination of waning population immunity, restored population mobility, and the widespread regional circulation of T1-lineage strains in the post-pandemic era. Viewed through a regional genomic-epidemiological lens, the trends in China and Japan are remarkably coherent, supporting the interpretation that the recent rise in type 1 *M. pneumoniae* represents an East Asia–wide resurgence rather than isolated, country-specific events.

## 6. Respiratory Viral Co-Infections and Microbiome Interactions

Before the COVID-19 pandemic, Diaz et al. [38] examined the frequency of co-infection with bacteria or viruses in *Mycoplasma pneumoniae*–positive specimens using a real-time PCR assay. They found that ≥1 additional pathogen was co-detected in approximately 60% of specimens, most frequently bacteria (*Haemophilus influenzae*, *Streptococcus pneumoniae*, *Staphylococcus aureus*, *Moraxella catarrhalis*), with co-detections being more common in children (65.5%) than in adults (34.2%) [38]. Interestingly, specimens with ≥3 co-detections in addition to *M. pneumoniae* were recorded only in patients younger than 17 years, whereas no such cases were observed in patients aged ≥18 years. These findings indicate that bacterial and viral pathogens frequently coexist in the respiratory tract. Spuesens et al. [22] further characterized the microbial context of *M. pneumoniae* carriage in children and reported that S. aureus was more frequently detected in asymptomatic than in symptomatic patients. They also noted that influenza A and B viruses, human metapneumovirus, and respiratory syncytial virus were predominantly detected in symptomatic children, whereas rhinovirus, bocavirus, and parainfluenza virus type 4 were more common in asymptomatic children [22].

In the post–COVID-19 era, Edouard et al. [32] reported that the concurrent presence of ≥1 respiratory virus (in order of frequency: rhinovirus, influenza A virus, respiratory syncytial virus, coronavirus OC43, influenza B virus, human metapneumovirus, etc.) was found in 36% of *M. pneumoniae*–positive patients, and that the prevalence of co-infection was significantly higher in children <5 years of age (51%) than in other age groups. Interestingly, co-infections were more common among patients diagnosed since 2023 (45.4%) than among those diagnosed during 2014–2022 (27%), suggesting a concomitant resurgence of respiratory viruses after the lifting of COVID-19 restrictions. Furthermore, Koenen et al. reported that *M. pneumoniae* carriage by qPCR is highly prevalent in young children suffering from recurrent infections who had lower alpha diversity of nasopharyngeal microbiota and a higher abundance of *Haemophilus influenzae* (OR 45.01) [25], suggesting a strong correlation between those pathogens in the upper respiratory tract (URT) microbiota.

Taken together, the advent of advanced molecular methods for detecting Mp (e.g., multiplex PCR, qPCR, microbiome analyses) has provided a new conceptual framework for understanding URT infections and community-acquired pneumonia involving co-infection with multiple bacterial and viral pathogens. Although the relative contribution of each respiratory pathogen has not been clearly defined, the host immune response, the presence of other pathogens, and the initial bacterial or viral load may collectively determine whether carriage progresses to clinical infection. A multidisciplinary approach is therefore warranted when Mp infection is suspected, taking into account regional epidemiology, patient age, household contact patterns, and especially concomitant respiratory viral infections, based on a careful clinical history.

## 7. Radiological Findings

**Figure 2:**
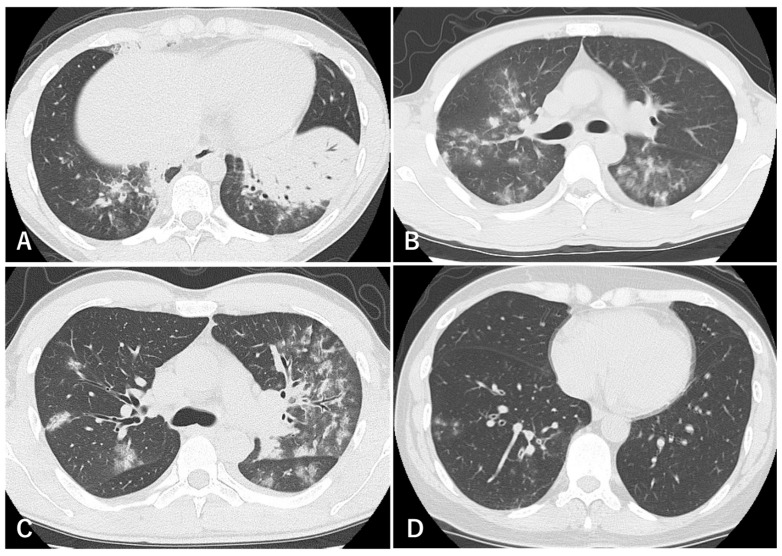
Representative thoracic computed tomography findings in adult *M. pneumoniae* pneumonia. Thoracic CT demonstrates dense consolidation in the left lower lobe with air bronchograms (**A**). Ground-glass opacities in a peribronchovascular distribution are shown in (**B**,**C**), accompanied by peribronchovascular thickening (**C**). Centrilobular nodules with bronchial wall thickening are shown in (**C**,**D**). GGO, ground-glass opacity. Reproduced from Ref. [44], with permission.

Chest radiographic findings in *Mycoplasma pneumoniae* pneumonia are variable and non-specific. In children, chest X-ray commonly shows consolidation as well as non-consolidative patterns such as localized reticulonodular opacities, parahilar peribronchial infiltrates, and patchy infiltrates; atelectasis and pleural effusion are less frequent. Accordingly, radiographs alone are insufficient for a definitive diagnosis and should be interpreted in conjunction with clinical and microbiological data [39]. In adults, radiographic findings are broadly similar; however, pleural effusion and atelectasis may be observed more often, with a tendency toward unilateral, lower-lobe–predominant involvement [40]. Lung ultrasound (LUS) is increasingly used as a point-of-care, radiation-free modality that is reproducible and can be repeated to monitor disease evolution, particularly in severe cases. LUS can identify and follow subpleural consolidations and pleural effusion, and several studies have suggested higher sensitivity than chest radiography for detecting pneumonia [41,42], although LUS findings are not pathogen-specific and should be interpreted alongside clinical and microbiological data. High-resolution CT (HRCT) better delineates airway-centered involvement in Mp pneumonia. Frequently reported CT abnormalities include bronchial wall and/or peribronchovascular thickening, centrilobular nodules (often presenting as a tree-in-bud pattern), ground-glass opacities, and areas of consolidation. Tanaka et al. reported radiology–pathology correlations in a murine model of *M. pneumoniae* infection, demonstrating that centrilobular nodules represent a key imaging hallmark of bronchiolitis [43]. In our representative cases (Figure 2) [44], consolidation is illustrated in Figure 2A; ground-glass opacities in Figure 2B,C; peribronchovascular thickening in Figure 2C; and centrilobular nodules in Figure 2C,D.

Overall, these CT findings tend to show a mid- to lower-lung predominance. Among them, airway-centered abnormalities—such as bronchial wall/peribronchovascular thickening and centrilobular nodules—are particularly helpful clues when Mp pneumonia is considered in the differential diagnosis [45].

## 8. Clinical Manifestations and Treatment Strategies for 2025

Most CAP guidelines [46,47,48] do not provide treatment recommendations specifically targeting *Mycoplasma pneumoniae* (Mp) pneumonia or macrolide-resistant Mp (MRMP). Instead, they describe broader strategies for covering atypical pathogens—including Mp, Legionella pneumophila, and Chlamydophila pneumoniae—as part of the initial empirical therapy for CAP, typically using a β-lactam agent combined with a tetracycline or macrolide, or alternatively a respiratory fluoroquinolone as monotherapy. However, they rarely address MRMP explicitly, and they do not provide tailored advice for settings where MRMP is highly prevalent, such as East Asia. In contrast, the JRS (Japanese Respiratory Society) guideline for the management of pneumonia in adults 2024 [49] clearly describes how to distinguish Mp pneumonia from other etiologies using simple clinical criteria (Table 3). If 5 or more of the 6 items are present, Mp pneumonia is suspected, whereas if 2 or fewer items are present, typical bacterial pneumonia is suspected. If 3 or 4 items are present, difficulty in differentiation or the possibility of mixed infection with both pathogens should be considered.

It is noteworthy that Mp pneumonia often has relatively poor chest auscultatory findings, similar to other cases of bronchopneumonia caused by *Haemophilus influenzae* or *Moraxella catarrhalis*. However, careful auscultation often reveals adventitious lung sounds, typically coarse crackles in the early phase of illness and fine crackles a few days after disease onset (Supplementary Audio S1: coarse crackles with rhonchi). In severe Mp pneumonia, particularly in patients with a productive cough, rhonchi or squawks may be present due to edematous changes in the bronchial wall (Supplementary Audio S2: squawk and rhonchi), whereas polyphonic wheezes resembling those of an asthma attack are uncommon [50].

Corresponding recordings can be accessed as Supplementary Audios S1 and S2 and via the QR codes shown in Supplementary Figure S1.

Importantly, the post-pandemic resurgence has been consistently associated with higher case volume; however, whether it also reflects increased clinical severity remains less clear. Comparisons of severity before versus after the pandemic are challenging because hospitalization, ICU admission, and mortality are not uniformly captured across surveillance systems, and case ascertainment and testing practices have changed over time. Standardized, age-stratified outcome reporting (including hospitalization, ICU admission, and mortality) will be essential to determine whether post-pandemic outbreaks represent a shift in disease severity or primarily a rebound in incidence.

Extrapulmonary manifestations of *Mycoplasma pneumoniae* (Mp) infection are diverse and include central nervous system (CNS) diseases such as encephalitis, meningoencephalitis, aseptic meningitis, myelitis, polyradiculitis, cerebellar ataxia, psychosis, Guillain–Barré syndrome, and peripheral neuropathy; cardiovascular diseases such as pericarditis, endocarditis, and myocarditis; dermatologic diseases including Stevens–Johnson syndrome, erythema multiforme, erythema nodosum, anaphylactoid purpura, and acute urticaria; hematologic diseases such as autoimmune hemolytic anemia (cold agglutinin disease), hemophagocytic syndrome, disseminated intravascular coagulation, and thrombocytopenic purpura. In addition, *Mycoplasma pneumoniae*–induced rash and mucositis (MIRM) is increasingly recognized as a characteristic mucocutaneous entity associated with Mp infection, particularly in children and young adults [51]. MIRM is typically characterized by prominent mucositis (oral/ocular/genital) with comparatively sparse skin lesions and is considered distinct from classic Stevens–Johnson syndrome/toxic epidermal necrolysis and erythema multiforme. Other inflammatory and organ-specific manifestations have also been reported, including conjunctivitis, iritis, uveitis, arthritis, and otitis media.

Neurologic complications of *Mycoplasma pneumoniae* infection typically develop 2–14 days after the onset of respiratory symptoms, that is, approximately 1–2 weeks after the initial infection. The presence of these extrapulmonary syndromes itself reflects the interaction between Mp and the host immune system. Notably, dermatologic involvement is observed in approximately 7% of patients [44], and extrapulmonary disease may occur in the absence of Mp pneumonia or even without any respiratory symptoms. CNS manifestations occur in fewer than 1 per 1000 cases and are most often reported in hospitalized patients; their pathogenesis remains unclear but is thought to involve a combination of direct invasion, toxin production, and autoimmune mechanisms such as autoantibody formation.

### 8.1. Antibiotic Therapy

Once Mp pneumonia is suspected, macrolide therapy (azithromycin, clarithromycin, or erythromycin) is considered first-line treatment. Clinical response should be reassessed within 48–72 h after initiation, focusing on defervescence and improvement in respiratory symptoms and/or inflammatory markers. However, if fever persists for 48–72 h after initiation of macrolide therapy, MRMP should be suspected and the treatment changed to minocycline or a respiratory fluoroquinolone (lascufloxacin, levofloxacin, or ciprofloxacin). Even in China, where the macrolide resistance rate exceeds 90.0%, consensus reports still recommend macrolides as first-line therapy [52]. In practice, escalation is particularly important in patients with progressive radiographic involvement, persistent hypoxemia, or worsening systemic inflammation despite macrolide therapy. However, for children aged ≥8 years with MRMP, doxycycline or minocycline is recommended. In younger children (<8 years), respiratory fluoroquinolones may be considered for selected severe MRMP pneumonia when clinical response to macrolides is inadequate, taking into account local guidance and antimicrobial stewardship [52]. These age-stratified recommendations illustrate the clinical dilemma: the pathogens most likely to cause Mp pneumonia are concentrated in younger age groups, where concerns about dental staining (tetracyclines) and cartilage toxicity (fluoroquinolones) are most acute.

### 8.2. Adjunctive Corticosteroids

Another area of ongoing debate is the role of adjunctive corticosteroid therapy. Systemic steroid treatment as add-on therapy may improve the outcome of severe Mp pneumonia, but its effects remain controversial. Thus, corticosteroids should not be considered routine therapy; rather, they may be reserved for selected severe or fulminant cases in which an excessive host immune response is suspected despite appropriate antimicrobial coverage. Izumikawa et al. reported that the majority of human patients with fulminant Mp pneumonia showed improvement in respiratory function within 3–5 days after initiation of steroid treatment [53]. The exact pathogenesis of fulminant Mp pneumonia remains unclear, but proposed mechanisms include a delayed hypersensitivity reaction to Mp and the contribution of delayed antibiotic administration to disease progression. The pathophysiology of Mp pneumonia is thought to be mainly associated with an excessive host immune response to Mp, which can lead to serious disease despite treatment with effective antimicrobial agents. From this viewpoint, the Japanese Society of Mycoplasmology has stated that systemic administration of corticosteroids may be effective in such patients. In particular, systemic corticosteroids are expected to be beneficial in patients with severe pneumonia characterized by fever lasting ≥7 days and a lactate dehydrogenase level >480 IU/L [26], with methylprednisolone 500–1000 mg/day for 3–5 days being a representative regimen in patients with respiratory failure. When steroids are used, careful reassessment for bacterial co-infection and close monitoring of clinical response are essential.

## 9. Future Directions: What Clinicians and Researchers Should Address Now

Taken together, the post-pandemic resurgence of *Mycoplasma pneumoniae* underscores that children are not only the most visibly affected group but also the principal reservoir sustaining community transmission. For clinicians, this means that careful age-stratified diagnosis, early recognition of Mp pneumonia, and timely adaptation of therapy in the setting of MRMP are no longer optional refinements but essential components of CAP management. The clinical significance of respiratory viral co-infection for disease severity remains to be clarified in future studies. The most immediate priority is to translate complex genomic and epidemiological signals into simple, age-stratified decision pathways. In high-MRMP prevalence settings, some regional expert groups consider earlier escalation in selected cases when clinical response is absent after 48–72 h; however, this approach should be applied within local stewardship policies and age-specific safety guidance and may not be generalizable to settings with low MRMP prevalence. Prospective studies are needed to establish standardized, age-stratified decision pathways for MRMP in children, including comparative effectiveness and safety of second-line regimens and the role of rapid resistance-informed diagnostics.

Long-term prevention. There is currently no licensed vaccine for *M. pneumoniae*, and prevention therefore remains largely reactive, centered on case recognition, testing, and treatment. Although vaccine development is conceptually attractive, progress has been limited and key hurdles include defining protective immunity and identifying safe, broadly protective antigens that can induce durable mucosal responses. In the interim, more proactive prevention will likely depend on strengthening age-stratified surveillance, rapid diagnostics, and antimicrobial-resistance monitoring, alongside pragmatic infection-prevention measures during school/community outbreaks (e.g., early case identification, guidance on return-to-school, and targeted mitigation during peaks).

Limitations. Because this is a narrative review, we did not apply a predefined systematic search strategy; accordingly, parts of the discussion reflect expert synthesis of the available evidence. In addition, published MRMP surveillance is geographically uneven; for example, a global meta-analysis found no published data from the WHO African region, underscoring that apparent regional gaps likely reflect under-ascertainment and reporting heterogeneity rather than absence of circulation. Finally, regional differences in diagnostic resources and drug availability limit the generalizability of a single management approach across all settings. Healthcare disparities and resource constraints may limit access to rapid molecular diagnostics and certain second-line agents in some settings; thus, implementation of recommended approaches should be adapted to local capacity and stewardship frameworks.

In summary, key takeaways for clinicians are as follows:

- Why now: The post-pandemic resurgence of *Mycoplasma pneumoniae* (Mp) has increased the pre-test probability of Mp pneumonia—especially in school-aged children—and is reshaping outpatient CAP workflows.
- Why children matter: Children likely serve as a major reservoir sustaining community transmission; age-stratified diagnostic and therapeutic pathways are therefore clinically relevant.
- How to diagnose: No single test is definitive. NAATs provide the most actionable early confirmation, whereas serology and culture are more useful for retrospective confirmation and surveillance, respectively (Table 1).
- How to treat: In high macrolide-resistance settings, persistent fever or lack of clinical improvement within 48–72 h on macrolides should prompt reassessment and consideration of age-appropriate alternative therapy.
- What’s next: Linking molecular epidemiology (genotypes/resistance markers) to simple bedside decision pathways is a near-term priority for both stewardship and patient outcomes.

## Figures and Tables

**Table 1:** Practical comparison of diagnostic modalities for *Mycoplasma pneumoniae* infection.

Modality	Sample	Turnaround Time *	Key Advantages	Key Limitations/Pitfalls	Recommended Clinical Use	Ref.
Culture (PPLO medium)	Respiratory specimen (e.g., throat/nasopharyngeal swab, sputum)	1–3 (up to 4) weeks	Reference method; enables isolate-based downstream analyses (e.g., phenotypic susceptibility testing, molecular epidemiology)	Too slow for rapid decisions; limited availability; technically demanding; yield may be reduced by prior antibiotics	Surveillance/research, outbreak investigation, or when an isolate is specifically required	[12,26]
Serology (IgM/IgG; CF/PA/ELISA)	Serum	Same day–2 days (paired sera: 2–4 weeks)	Widely accessible; useful for retrospective confirmation; paired sera can support acute infection	Single acute-phase results are difficult to interpret; timing dependent; variable IgM response; potential cross-reactivity; not optimal for early therapy decisions	When NAAT is unavailable/negative, but suspicion persists; paired sera for confirmation/epidemiology	[12,26]
NAAT (singleplex PCR/real-time PCR)	Oropharyngeal/throat swab, nasopharyngeal swab, BAL, tracheal aspirate specimen, sputum(when available)	Hours–1 day	Rapid, sensitive direct detection; suitable for respiratory specimens; supports early decision-making	Institution-dependent assay availability/workflow; NAAT positivity does not always establish causality (possible carriage, especially in children); assay/specimen variability; typically, does not provide phenotypic susceptibility	First-line test when available; interpret with clinical context and timing from symptom onset	[12,26]
LAMP	Oropharyngeal/throat swab, sputum (kit-/platform-dependent)	~30–90 min	Rapid; isothermal workflow; potentially deployable with less complex equipment	Performance depends on kit/specimen; limited add-on information (e.g., resistance) unless specifically designed; still requires appropriate sampling/handling	When rapid testing is needed in settings without full PCR infrastructure; triage/outpatient/smaller facilities	[12,26]
Q-probe PCR	Oropharyngeal/throat swab, sputum (kit-/platform-dependent)	~1–2 h (platform dependent)	Rapid NAAT format; some assays can simultaneously detect macrolide-resistance–associated mutations (e.g., 23S rRNA) to inform early optimization	Availability and cost; mutation coverage is assay-specific; requires careful interpretation (detection does not necessarily indicate causality)	High-MRMP settings or when resistance-aware decision-making is prioritized (e.g., persistent fever on macrolides)	[26]
Multiplex PCR panel (syndromic testing)	Nasopharyngeal swab/sputum/saliva (panel-/platform-dependent)	~1–2 h (often same day)	Broad detection of viruses/bacteria (including Mp) from one specimen; helpful when co-pathogens may affect management	Target performance varies; cost; detection does not necessarily indicate causality; may detect colonizers; stewardship concerns (risk of unnecessary antibiotics)	Moderate–severe CAP, immunocompromised hosts, diagnostic uncertainty, or when co-infection is likely/clinically relevant	[27]
Antigen test (immunochromatographic)	Oropharyngeal/throat or nasopharyngeal swab (kit-dependent)	~10–30 min	Very rapid; simple workflow; may assist front-line screening	Lower sensitivity than NAAT; false negatives; mucus-rich/highly viscous specimens may cause nonspecific reactivity and occasional false positives; confirm with NAAT when discordant with clinical suspicion	Rapid screening when NAAT is not immediately available; confirm with NAAT and/or paired serology if suspicion remains high	[12,26]

Abbreviations: CAP, community-acquired pneumonia; CF, complement fixation; PA, passive agglutination; ELISA, enzyme-linked immunosorbent assay; LAMP, loop-mediated isothermal amplification; MRMP, macrolide-resistant *Mycoplasma pneumoniae*; Mp, *Mycoplasma pneumoniae*; NAAT, nucleic acid amplification test; PCR, polymerase chain reaction; PPLO, pleuropneumonia-like organism. * Turnaround time may vary depending on institutional workflow and platform.

**Table 2:** Major regional outbreaks (2023–2025) and MRMP rates.

Country/Region	Period	Age Group	MRMP Rate	Ref.
China—Wuhan	2023	Children	84.52%	[28]
China—Beijing	Late 2023	Children	100%	[29]
China—Beijing —Baoding	November 2023–February 2024	Children	99.41%	[30]
Denmark—National	October–December 2023	Children Adolescents Adults	3.6%	[31]
France—Marseille	2023–2024	Children Adolescents Adults	Not examined	[32]
Switzerland—Southern region	November 2023 onward	Adults	Not examined	[33]
Japan	2024	Children	54.1%	[34]

Abbreviations: MRMP, macrolide-resistant *Mycoplasma pneumoniae*.

**Table 3:** Characteristics of patients with *Mycoplasma pneumoniae* pneumonia.

Items Used for Diagnosis
Under 60 y of age
No or minor underlying disease
Stubborn cough
Poor chest auscultatory findings
No sputum or etiological agent identified by rapid diagnosis *
A peripheral white blood cell count <10,000/μL

* Excluding cases with a positive Mycoplasma antigen or genetic (PCR) test. Adapted from Ref. [49].

## Data Availability

No new data were created or analyzed in this study.

## Supplementary Materials

The following supporting information can be downloaded at: https://www.mdpi.com/article/10.3390/jcm15041644/s1, Figure S1. QR codes linking to lung sound recordings. (A) Supplementary Audio S1: Coarse crackles with rhonchi in *Mycoplasma pneumoniae* pneumonia. The QR code links directly to the internal company webpage for interactive playback. The corresponding audio file is additionally archived on Zenodo as the citable source (Concept DOI: https://doi.org/10.5281/zenodo.18605227, accessed on 8 January 2026). The authors retain all rights to the original audio recording. (B) Supplementary Audio S2: Squawk and rhonchi in severe *Mycoplasma pneumoniae* pneumonia. The QR code links directly to the internal company webpage for interactive playback. The corresponding audio file is additionally archived on Zenodo as the citable source (Concept DOI: https://doi.org/10.5281/zenodo.18605339, accessed on 8 January 2026). The authors retain all rights to the original audio recording. Each recording can be accessed via the corresponding QR code, and listening with earphones/headphones is recommended.
