# Supplementary figures and images for: The Post-Pandemic Return of Mycoplasma pneumoniae: Why Children Matter and What Clinicians Should Know
Source: J Clin Med. 2026 Feb 22;15(4):1644. doi: 10.3390/jcm15041644 (PMC12942111; doi:10.3390/jcm15041644)

## Supplemental FigureS1

Audio S1

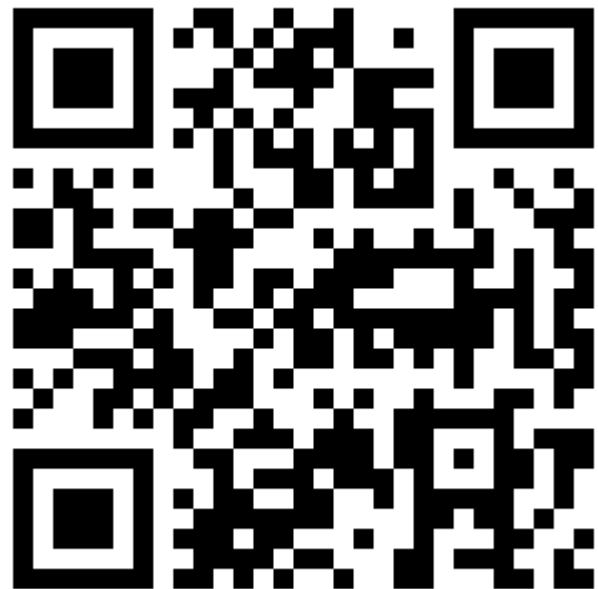

Audio S2

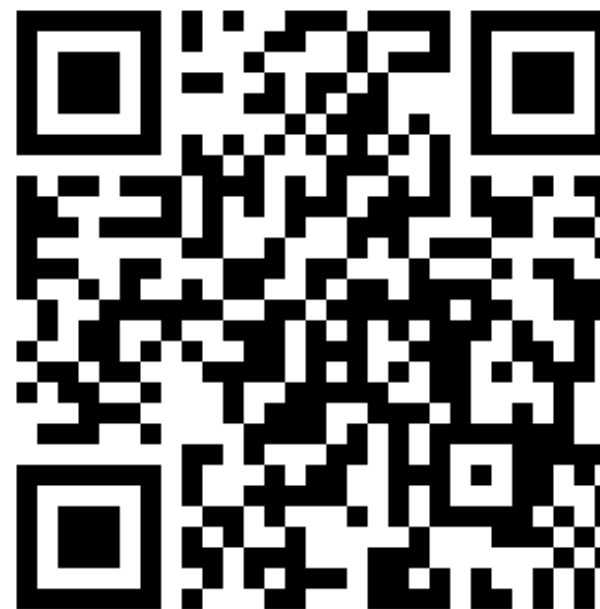

Supplement: Supplementary file 1 [file jcm-15-01644-s001.zip › jcm-4116940-supplementary.pdf]
